# Short-term single treatment of chemotherapy results in the enrichment of ovarian cancer stem cell-like cells leading to an increased tumor burden

**DOI:** 10.1186/1476-4598-12-24

**Published:** 2013-03-27

**Authors:** Khalid Abubaker, Ardian Latifi, Rod Luwor, Simon Nazaretian, Hongjian Zhu, Michael A Quinn, Erik W Thompson, Jock K Findlay, Nuzhat Ahmed

**Affiliations:** 1 Women’s Cancer Research Centre, Royal Women’s Hospital, Victoria 3052, Australia; 2 Department of Surgery, University of Melbourne, St Vincent Hospital, Victoria, Australia; 3 Department of Surgery, University of Melbourne, Royal Melbourne Hospital, Victoria, 3052, Australia; 4 Department of Anatomical Pathology, Royal Women’s Hospital, Victoria 3052, Australia; 5 Department of Obstetrics and Gynaecology, University of Melbourne, Victoria 3052, Australia; 6 St Vincent’s Institute, Victoria, 3065, Australia; 7 Prince Henry’s Institute of Medical Research, Victoria 3168, Australia

**Keywords:** Ovarian carcinoma, Cancer stem cell, Metastasis, Ascites, Chemoresistance, Recurrence

## Abstract

Over 80% of women diagnosed with advanced-stage ovarian cancer die as a result of disease recurrence due to failure of chemotherapy treatment. In this study, using two distinct ovarian cancer cell lines (epithelial OVCA 433 and mesenchymal HEY) we demonstrate enrichment in a population of cells with high expression of CSC markers at the protein and mRNA levels in response to cisplatin, paclitaxel and the combination of both. We also demonstrate a significant enhancement in the sphere forming abilities of ovarian cancer cells in response to chemotherapy drugs. The results of these *in vitro* findings are supported by *in vivo* mouse xenograft models in which intraperitoneal transplantation of cisplatin or paclitaxel-treated residual HEY cells generated significantly higher tumor burden compared to control untreated cells. Both the treated and untreated cells infiltrated the organs of the abdominal cavity. In addition, immunohistochemical studies on mouse tumors injected with cisplatin or paclitaxel treated residual cells displayed higher staining for the proliferative antigen Ki67, oncogeneic CA125, epithelial E-cadherin as well as cancer stem cell markers such as Oct4 and CD117, compared to mice injected with control untreated cells. These results suggest that a short-term single treatment of chemotherapy leaves residual cells that are enriched in CSC-like traits, resulting in an increased metastatic potential. The novel findings in this study are important in understanding the early molecular mechanisms by which chemoresistance and subsequent relapse may be triggered after the first line of chemotherapy treatment.

## Introduction

Epithelial ovarian cancer (EOC) is the fifth most common cancer among women and is the leading cause of death among gynaecological cancers. Over 80% of women with EOC are diagnosed at a late-stage with dissemination of tumor implants throughout the peritoneal cavity [1]. The combination of cisplatin and paclitaxel based chemotherapy was introduced as a first line of treatment for the clinical management of advanced-stage ovarian cancer patients nearly 17 years ago [2]. Cisplatin is a DNA strand cross-linking drug that generates DNA damage leading to the activation of cyclin-dependent kinase inhibitors such as p21 and wee1/mik1, which subsequently arrest cells in either G1 or G2 phase [3]. Resistance to cisplatin has been associated with increased glutathione and metallothionein levels, decreased drug uptake, increased DNA repair (due to enhanced expression of excision repair enzymes) and the tolerance of the formation of platinum-DNA adducts [4]. The status of p53 mutation plays a significant role in DNA repair, proliferative arrest and apoptosis and there is a correlation between cancer cell p53 status and cisplatin sensitivity [5,6]. Paclitaxel on the other hand, is a mitotic inhibitor that promotes the formation and stabilization of microtubules leading to a cell cycle block at the metaphase to anaphase transition [7]. In contrast to cisplatin, the cytotoxic effect of paclitaxel is independent of p53 status [8] and alterations in β-tubulin isotypes have been associated with paclitaxel resistance in cancer cells [8]. Both cisplatin and paclitaxel through distinct molecular mechanisms trigger an apoptotic cascade resulting in the death of the majority of ovarian cancer cells. In spite of this, approximately 80% of ovarian cancer patients experience incurable recurrent cancer within 6–20 months post-chemotherapy [1] as a consequence of the survival of a very small percentage of chemotherapy resistant residual tumor cells which facilitate the development of recurrent progressive disease [1]. Concerted research efforts to tackle the failure of combination chemotherapy have resulted in no effective salvage strategies for the last 17 years [9]. Hence, there is an increasing pressure to seek alternative approaches, which has resulted in the use of combinations of drugs that usually belong to the platinum or taxane families [9]. These alternative drug combinations have provided temporary hope to the patients but have had no clinically effective outcome [9]. To establish an effective treatment protocol for advanced-stage ovarian cancer patients a systematic approach is needed to understand responses of ovarian cancer cells to platinum and taxane-based drugs, individually and in combination. *In vivo* experiments initially with each drug treatment will result in insights into the molecules that facilitate the evasion of chemotherapy-associated cytotoxicity against each individual drug and the subsequent re-growth of tumour cells as recurrent tumor masses. This is particularly important for a large proportion of chemorefractory ovarian cancer patients who are resistant to platinum-based drugs and are normally prescribed taxane-based treatment. On the other hand, some ovarian cancer patients respond badly towards taxane-based drugs and develop serious side effects, in which case they are prescribed platinum-based treatment.

We and others have recently demonstrated an association between chemoresistance and the acquisition of epithelial mesenchymal transition (EMT) and CSC-like phenotypes in cancer [10-12] and found chemoresistant recurrent ovarian tumors to be enriched in CSCs and stem cell pathway mediators, suggesting that CSCs may contribute to recurrent disease [13,14]. The first involvement of stem cells in ovarian cancer was reported in the ascites of an ovarian cancer patient, derived from a single cell that could sequentially propagate tumors over several generations [15]. CSCs have also been isolated from ovarian cancer cell lines based on their abilities to differentially efflux the DNA binding dye Hoechst 33342 [16]. This population of cells termed the ‘side population’ (SP) displayed the classical stem cell property in tumorigenicity assays. More recently, a population of normal murine OSE [17] have been identified to have putative stem cell characteristics indicating that these may be the originators of CSCs in the ovaries. Few other recent reports have shown the presence of CSCs in ovarian tumors as well as in patients’ ascites [18-20]. CSCs in these studies were reported to be resistant to conventional chemotherapy and were able to recapitulate *in vivo* the original tumor suggesting that these CSCs control self-renewal as well as metastasis and chemoresistance.

In this study, we demonstrate that a short-term single exposure of chemotherapy (cisplatin, paclitaxel or both in combination) treatment induced in surviving ovarian cancer cells a CSC-like profile which was independent of the type of chemotherapy and the associated cytotoxicity. We further demonstrate that chemotherapy surviving residual cells were able to generate tumors with greater capacity (tumor burden) than control untreated cells, and that they retained their inherent CSC-like profile in tumor xenografts. These novel findings emphasize the need to understand the CSC-like phenotype of ovarian tumors which may arise after the first line of chemotherapy treatment and may be crucial in facilitating the aberrant events leading to recurrent disease.

## Methods and materials

### Cell lines

The human epithelial ovarian cancer line OVCA 433 was derived from the ascites of an ovarian cancer patient and generously provided by Dr Robert Bast Jr. (MD Anderson Cancer Centre, Houston, TX). The cell line was grown as described previously [11]. The human ovarian HEY cell line was derived from a peritoneal deposit of a patient diagnosed with papillary cystadenocarcinoma of the ovary [21]. The cell line was grown as described previously [22].

### Antibodies and reagents

Monoclonal and polyclonal antibodies against CD44, CD24, CD117, CD133, were obtained from Millipore (Melbourne, Australia). Monoclonal antibodies against excision repair complement complex 1 (ERCC1) and β-tubulin isotype III were obtained from Sapphire Biosciences and Sigma Aldrich (Melbourne, Australia). Polyclonal antibody against EpCAM was obtained from Cell Signalling Technology (Beverly, MA, USA). Antibodies against cytokeratin 7 (cyt7), Ki67, CA125, E-cadherin, vimentin, Oct4 and CD117 (c-Kit) used for immunohistochemistry were obtained from Ventana (Roche, Arizona, USA).

### Treatment of ovarian cancer cells with cisplatin, paclitaxel and combination of both

Ovarian cancer cell lines OVCA 433 and HEY were treated with cisplatin and paclitaxel concentrations at which 50% growth inhibition was obtained (GI50) for 3–5 days. OVCA 433 cells were treated with cisplatin (5 μg/ml) for five days, paclitaxel (2 ng/ml) and combination (2.5 μg/ml of cisplatin and 1 ng/ml of paclitaxel) for three days. HEY cells were treated with cisplatin (1 μg/ml) five days, paclitaxel (1 ng/ml) and combination (1 μg/ml of cisplatin and 1 ng/ml of paclitaxel) for three days. For combination treatment, samples were screened for response to different combination of drug treatments and the concentration of combination treatment which gave the GI50 value while maintaining the enhancement in resistant phenotype (ERCC1 and β-tubulin expression) and cancer stem cell marker expression was chosen for experiments.

### Immunofluorescence analysis

Immunofluorescence analysis of ERCC1 and β-tubulin isotype III was performed as described previously [13]. Images were captured by the photo multiplier tube (PMT) using the Leica TCS SP2 laser, and viewed on a HP workstation using the Leica microsystems TCS SP2 software. The mean fluorescence intensity was quantified using Cell-R software (Olympus Soft Imaging Solutions).

### Flow cytometric analysis

Flow cytometry was performed as described previously [23]. Briefly, untreated or chemotherapy treated cells were collected and rinsed twice with phosphate buffered saline (PBS). 10^6^ cells were incubated with primary antibody for 1 hr at 4°C and excess unbound antibody was removed by washing twice with PBS. Cells were stained with secondary antibody conjugated with phycoerythrin for 20 minutes at 4°C, washed twice with PBS and then resuspended in 0.5 ml PBS prior to FACScan analysis. In each assay background staining was detected using an antibody-specific IgG isotype. All data were analysed using Cell Quest software (Becton-Dickinson, Bedford, MA, USA). Results are presented as histogram overlay.

### Sphere forming assay

The sphere forming ability of untreated and chemotherapy treated OVCA 433 cells and HEY cells were determined as described previously [11]. The sphere forming ability of the cells was photographed over 21 days using a phase contrast microscope (Axiovert 100, Zeiss, Germany) and assessed with the DeltaPix Viewer software (Denmark). Cellular aggregates with a diameter larger than 50 μm were classified as ‘spheres’.

### RNA extraction and Real Time-PCR

RNA extractions were performed using Trizol (Life Technologies, USA) using the Qiashredder and RNeasy kits (QIAGEN, Australia) according to the manufacturer’s instructions. The concentration and purity of RNA was determined using spectrophotometry (Nanodrop ND-1000 spectrophotometer, Thermo Scientific, USA) and 0.5 μg of RNA was used for cDNA synthesis. cDNA synthesis was performed using Superscript VILO (Invitrogen, Australia) according to manufacturer’s instructions. Quantitative determination of mRNA levels of various genes was performed in triplicate using SYBR green (Applied Biosystems, Australia) as described previously [13]. The primers for Oct-4A, Nanog, CD44, CD117, EpCAM have been described previously [11].

### Animal studies

#### Animal ethics statement

This study was carried out in strict accordance with the recommendations in the Guide for the Care and Use of the Laboratory Animals of the National Health and Medical Research Council of Australia. The experimental protocol was approved by the Ludwig/Department of Surgery, Royal Melbourne Hospital and University of Melbourne’s Animal Ethics Committee (Project-006/11), and was endorsed by the Research and Ethics Committee of Royal Women’s Hospital Melbourne, Australia.

#### Animal experiments

Female Balb/c nu/nu mice (age, 6–8 weeks) were obtained from the Animal Resources Centre, Western Australia. Animals were housed in a standard pathogen-free environment with access to food and water.

HEY cells were treated with cisplatin and paclitaxel as described previously. 5x10^6^ residual cisplatin or paclitaxel surviving cells treated for 4 days were injected intraperitoneally (ip) in nude mice. Mice were inspected weekly and tumor progression was monitored based on overall health and body weight until one of the pre-determined endpoints was reached. Endpoint criteria included loss of body weight exceeding 20% of initial body weight, anorexia, general patterns of diminished well-being such as reduced movement and lethargy resulting from lack of interest in daily activities. Mice were euthanized and organs (liver, stomach, lungs, gastrointestinal tract, pancreas, uterus, skeletal muscle, colon, kidney, peritoneum, ovaries and spleen) and solid tumors were collected for further examination. Metastatic development was documented by a Royal Women’s Hospital pathologist according to histological examination (H & E staining) of the organs.

### Immunohistochemistry of mouse tumors

For immunohistochemistry, formalin fixed, paraffin embedded 4 μm sections of the xenografts were stained using a Ventana Benchmark Immunostainer (Ventana Medical Systems, Inc, Arizona, USA). Detection was performed using Ventana’s Ultra View DAB detection kit (Roche/Ventana, Arizona, USA) using the method described previously [24]. Briefly, tumor sections were dewaxed with Ventana EZ Prep and endogenous peroxidase activity was blocked using the Ventana’s Universal DAB inhibitor. Primary antibodies against Oct4, Ki67, E-cadherin, vimentin, CA125, cytokeratin 7 and CD117 (c-Kit) were diluted according to the instruction provided by the manufacturer. The sections were counter stained with Ventana Haematoxylin and Blueing Solution. Immunohistochemistry images were taken using Axioskop 2 microscope, captured using a Nikon DXM1200C digital camera and processed using NIS-Elements F3.0 software.

### Statistical Analysis

Student’s t-test was used for the statistical analyses of sphere formation and qPCR analysis. Data are presented as mean ± SEM. A probability level of p < 0.05 was adopted throughout to determine statistical significance. Treatment groups were compared with the control group using one way- ANOVA and Dunnett’s Multiple Comparison post-tests.

## Results

### Chemotherapy induced morphological changes in ovarian cancer cell lines

**Figure 1:**
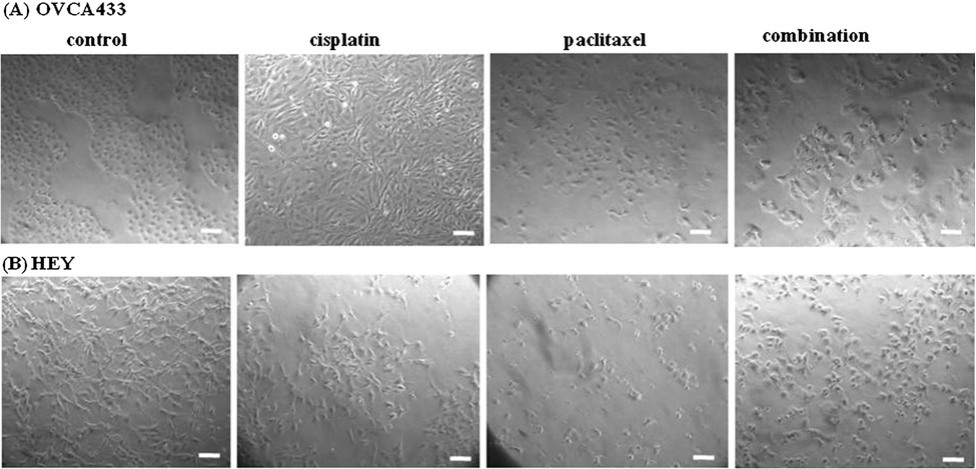
**Morphological features of OVCA433 and HEY cell lines under normal culture conditions (control) and after treatment with chemotherapy.** (**A**) OVCA 433 cell line was treated with cisplatin (5 μg/ml) for five days, paclitaxel (2 ng/ml) for three days and combination of cisplatin and paclitaxel (2.5 μg/ml cisplatin and 1 ng/ml paclitaxel) for three days. (**B**) HEY cell line was treated with cisplatin (1 μg/ml) for five days, paclitaxel (1 ng/ml) for three days and combination of cisplatin and paclitaxel ( 1 μg/ml cisplatin and 1 ng/ml paclitaxel) for three days. The images were assessed by phase contrast microscope. Magnification- 100x, scale bar = 10 μm.

Treatment with cisplatin resulted in a loss of cell polarity in epithelial OVCA 433 cells and was consistent with fibroblast-like spindle-shaped morphology in treated cells as described previously [11,12] (Figure 1A). Due to the inherent mesenchymal morphology of HEY cells, changes in mesenchymal morphology in response to cisplatin treatment was not prominent in HEY cells (Figure 1B). On the other hand, treatment with paclitaxel resulted in the appearance of rounded epitheloid cells within three to five days in both cell lines (Figures 1A-B). The change to epithelial morphology in response to paclitaxel was more prominent in HEY than in OVCA 433 cells, due to their initial mesenchymal appearance. Some HEY cells seemed to undergo phenomenal cellular enlargement which was up to five-fold (approximately 50 μm in diameter) more than the control untreated cells. This may be due to the formation of multi-nucleated cells in response to paclitaxel treatment which may result from the inhibition of the mitotic cycle at the metaphase to anaphase stage i.e. when the cell fails to divide into two daughter cells even though the distribution of centrosome/nucleosome for the daughter cells have occurred.

Morphological changes in response to cisplatin or paclitaxel were dose-dependent (data not shown). Cisplatin-induced morphological changes were evident at concentrations between 1–10 μg/ml (GI50 ~ 5 μg/ml) for OVCA 433 cells. However, HEY cells responded to much lower cisplatin concentration of 0.5-5 μg/ml (GI50 ~ 1 μg/ml) (Figures 1A-B). On the other hand, paclitaxel-induced epithelial morphology was evident at a concentration of 0.5-2.5 ng/ml (GI50 ~ 2 ng/ml) for OVCA 433 cells, and 0.1-2 ng/ml (GI50 ~ 1 ng/ml) for HEY cells. Similar change to epithelial morphology in clones of surviving cells, but to a greater extent than that observed with paclitaxel only, was evident after combination treatment (cisplatin + paclitaxel). Both OVCA 433 and HEY demonstrated discrete epithelial colonies and very few mesenchymal cells which were scattered in between epithelial cells (Figures 1A-B). Different concentrations of combination treatments were tried but as described previously [11] the drugs concentration at or below the GI50 value were used for further study.

### Chemotherapy induces the expression of cisplatin and paclitaxel resistant phenotypes

**Figure 2:**
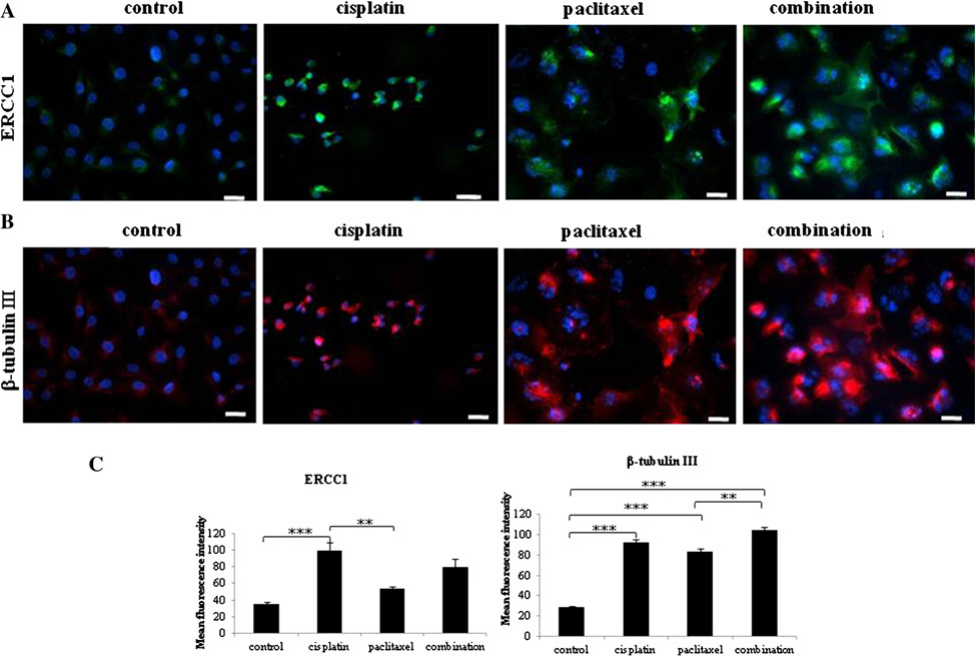
**Expression and immunolocalization of (A) ERCC1 and (B) β-tubulin isotype III in HEY cell line in response to cisplatin, paclitaxel and combination treatment.** The images were evaluated using mouse monoclonal (green) and rabbit polyclonal (red) antibodies as described in the Materials and methods section. Cellular staining was visualized using secondary Alexa 488 (green) and Alexa 590 (red) fluorescent labelled antibodies. Nuclear staining was visualized using DAPI (blue) staining. Images are representative of three independent experiments. Magnification 200x; scale bar = 10 μM. (**C**) The mean fluorescence intensity was quantified using Cell-R software (Olympus Soft Imaging Solutions). Significant variations between the groups are indicated by ** P < 0.01, *** P < 0.001.

In order to determine if the morphological changes induced by cisplatin and paclitaxel were consistent with the chemoresistant phenotype of the ovarian tumors as described previously [25,26], we evaluated the expression of ERCC1 and β-tubulin isotype III by cancer cells which survived cisplatin, paclitaxel and combination treatments using immunofluorescence. Compared to untreated control cells, enhanced expression of ERCC1 was evident in cisplatin, paclitaxel and combination treated HEY cells (Figure 2). Enhanced β-tubulin isotype III staining was also evident in HEY cells surviving cisplatin, paclitaxel and combination treatment (Figures 2A). In most of the cases, the same population of residual cells stained for ERCC1 and β-tubulin isotype III after the 3 treatments, suggesting cross resistance for cisplatin and paclitaxel in HEY cells. However, β-tubulin isotype III was more dominant in paclitaxel and combination treated cells (Figure 2B). The expression of ERCC1 was confined mainly to peripheral membranes in most cells and few cells displayed nuclear staining. In response to paclitaxel treatment an increase in the expression of β-tubulin isotype III was evident on the peripheral membrane as well as in the nucleus of the surviving cells (Figure 2B). However, there was more nuclear β-tubulin isotype III staining compared to membrane staining after combination treatment (Figure 2B). OVCA 433 cells demonstrated a similar ERCC1 and β-tubulin isotype III staining pattern (Additional file 1: Figure S1). Quantitative measurement of the expression of ERCC1 demonstrated significant enhancement in the expression of ERCC1 in both HEY and OVCA 433 cells in response to cisplatin treatment (Figure 2 and Additional file 1: Figure S1). The expression of ERCC1 was significantly higher in paclitaxel and combination treated OVCA 433 cells but was not evident in HEY cells under similar treatment conditions (Figure 2 and Additional file 1: Figure S1). On the other hand, β-tubulin isotype III expression was significantly higher in paclitaxel treated OVCA 433 and HEY cells. No change in β-tubulin isotype III expression was observed in cisplatin and combination treated OVCA 433 cells, while significant enhancement in the expression was observed in cisplatin and combination treated HEY cells compared to control untreated cells (Figure 2 and Additional file 1: Figure S1).

### Chemotherapy enhances the expression of CSC markers

**Figure 3:**
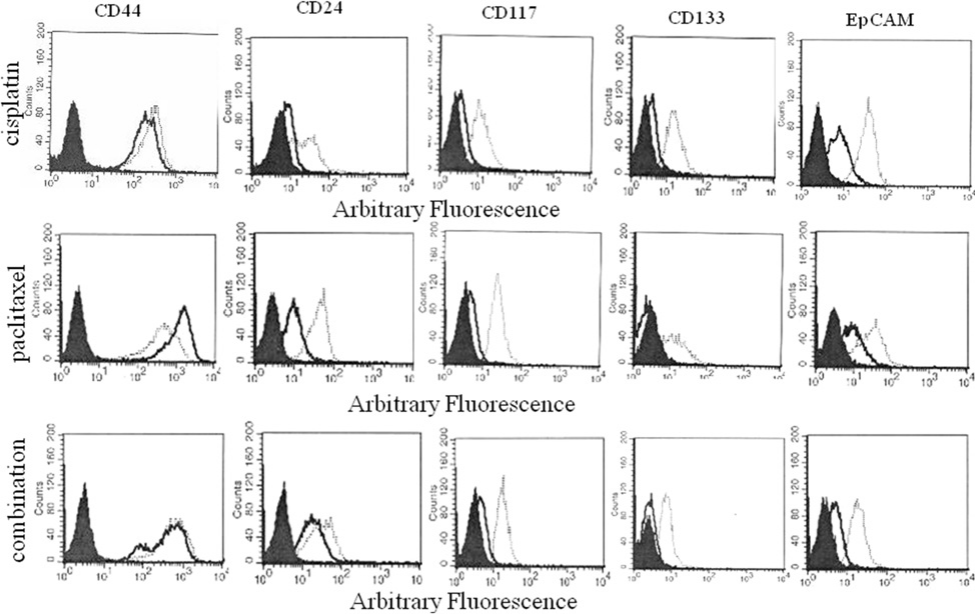
**The effects of cisplatin, paclitaxel and combination treatments on the expression of CSC-like markers in HEY cells.** Untreated or chemotherapy treated cells were incubated with either control IgG or relevant primary antibodies against the respective CSC-like markers followed by secondary goat anti-mouse IgG conjugated with phycoerythrin. The filled histogram in each figure is control IgG, black lines indicate protein expression in control cells while broken lines demonstrate protein expression in treated cells. Results are representative of 3–4 independent experiments.

Recently a CSC-like phenotype has been demonstrated in drug resistant ovarian cancer cell lines [16,27] and also in primary and metastatic ovarian cancer cells from patients [14,19,28]. In order to assess the status of this phenomenon in response to cisplatin or paclitaxel and combination chemotherapy treatments, we assessed the cell surface expression of some known CSC markers [18] by flow cytometry in OVCA 433 and HEY cells. Moderate to low expression of CD44, CD24, CD117, CD133 and EpCAM was evident by flow cytometry in OVCA 433 and HEY cells (Figure 3 and Additional file 2: Figure S2). The expression of CD24, CD117, CD133 and EpCAM increased in HEY cells with cisplatin, paclitaxel and combination treatments, while there was no change in the expression of CD44 in response to cisplatin and combination treatments (Figure 3). Paclitaxel treatment on the other hand, resulted in the decrease of CD44 expression in HEY cells. In OVCA 433 cells there was an increase in the expression of CD44, CD24, CD117, CD133 and EpCAM in response to cisplatin, paclitaxel and combination treatments (Additional file 2: Figure S2). However, the increase in CD44 was not pronounced in response to cisplatin.

**Figure 4:**
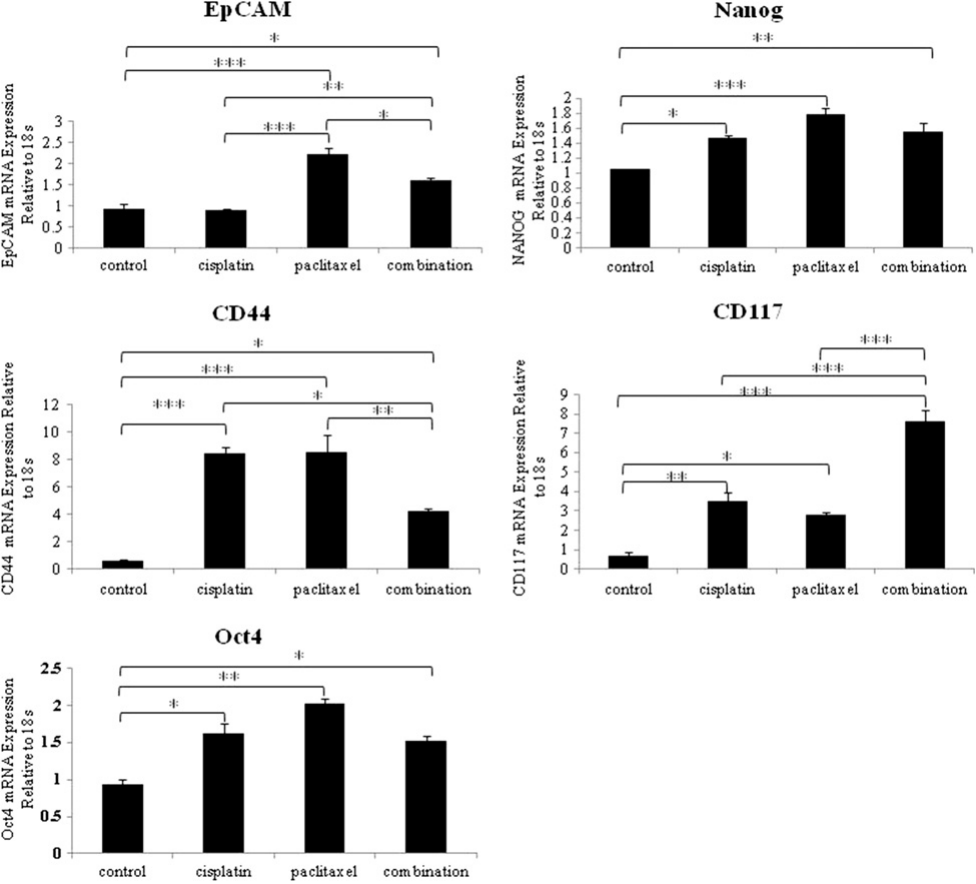
**mRNA expression of EpCAM, Nanog, CD44, CD117 and Oct4 in HEY cell line in response to chemotherapy treatments (cisplatin, paclitaxel and combination).** Cells were treated with or without chemotherapy, RNA was extracted, cDNA was prepared and qPCR was performed as described in the Materials and methods section. The resultant mRNA levels were normalized to 18S mRNA. The experiments were performed using four independent HEY samples in triplicate. Significant intergroup variations are indicated by *P <0.05, ** P < 0.01, *** P < 0.001.

The CSC-like profile of drug-treated ovarian cancer cells was further assessed at the mRNA level by qPCR (Figure 4). Significantly enhanced mRNA expression of CD44, EpCAM, CD117, Oct-4 and Nanog in response to paclitaxel and combination chemotherapy was observed in HEY cells (Figure 4). Although significant increases in the mRNA levels of CD44, CD117, Oct4 and Nanog were observed in response to cisplatin treatment, no enhancement in the expression of EpCAM was observed. Hence, the results obtained for EpCAM and CD44 in response to cisplatin treatment differed at the protein and mRNA levels (Figure 4). In OVCA 433 cells however, the mRNA expression of CD44, EPCAM, CD117, Oct4A and Nanog was significantly enhanced under all three treatment conditions compared to untreated controls (Additional file 3: Figure S3).

**Figure 5:**
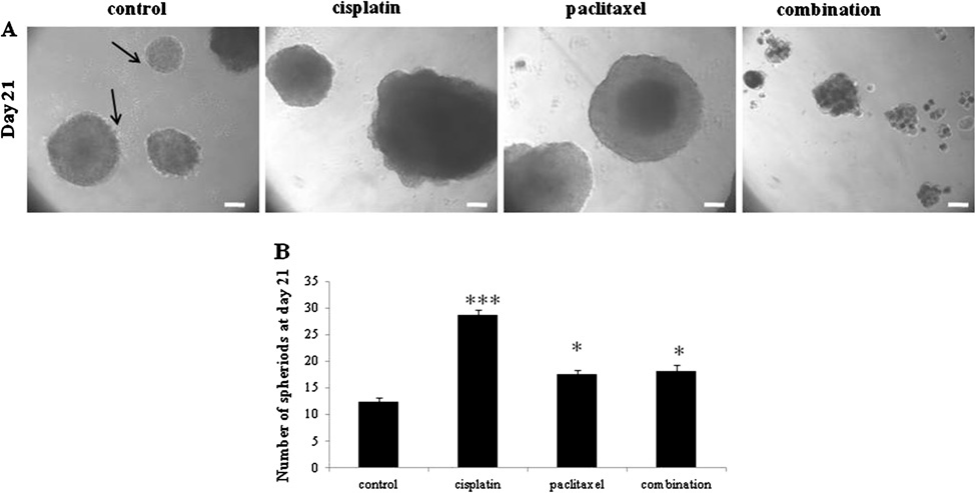
**Effects of chemotherapy on the sphere forming ability of HEY cells.** (**A**) The sphere-forming assay was performed on low attachment plates as described in the Material and methods section. The total number of spheres was counted in the 24 well plates after 21 days as described in the Methods and materials. The experiment was performed three times in triplicate. Images are representative of a section of a 24 well plate. Black arrows indicate disaggregating cells in control spheres in 21 days. Magnification 200x; scale bar = 10 µM. (**B**) Significantly different from control untreated cells indicated by *P<0.05, *** P<0.001.

As sphere formation has been described as an important feature for the survival of ovarian CSCs [15], we evaluated the sphere forming abilities of control, cisplatin, paclitaxel and combination treated HEY and OVC 433 cells (Figure 5 and Additional file 4: Figure S4). In long term cultures, control and chemotherapy treated cells demonstrated the ability to form spheres on low attachment plates (Figure 5 and Additional file 4: Figure S4). Within 21 days, the aggregates formed by cisplatin, paclitaxel and combination therapy treated cells took the shape of spheres with a defined outer rim and were significantly greater in numbers than control cells (Figure 5 and Additional file 4: Figure S4). However, majority of the spheres formed by paclitaxel-treated HEY cells were much bigger in size than the spheres generated from cisplatin or combination treated cells. This was due to the aggregation of relatively bigger multinucleated cells. Hence, the number of spheres with a diameter larger than 50 μm was less than the spheres of cisplatin or combination treated HEY cells in each field counted under the microscope (Figure 5). In response to combination treatment, cells produce viable spheres but these were smaller than spheres formed by either cisplatin or paclitaxel treated cells. This may be due to the mixture of epithelial and mesenchymal cells which may not have the inherent capacity to aggregate and form bigger spheres. Many cellular aggregates (spheres) formed from control untreated cells disaggregated within the 21 day time point but those formed by drug-treated cells persisted, suggesting that chemotherapy transformed residual cells have a greater ability to survive in anchorage independent conditions and are enriched in self-renewing capability compared to control untreated cells.

### Residual cancer cells after chemotherapy treatments exhibited metastatic and CSC-like features in nude mice

**Figure 6:**
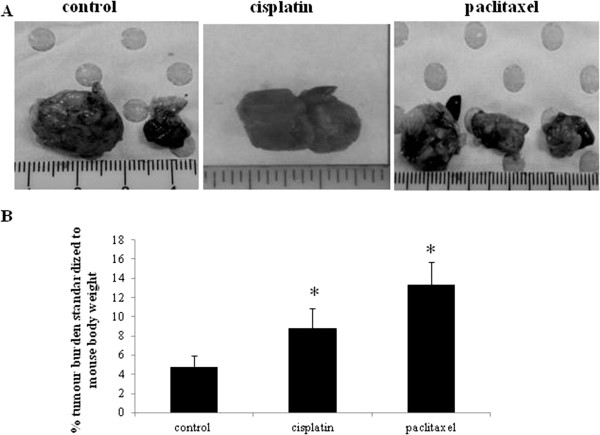
**Tumor burden of mice injected with untreated control and chemotherapy treated HEY cells.** (**A**) Total tumor burden obtained from mice 6 weeks after ip injection of control and chemotherapy treated HEY cells. 5x10^6^ cells were inoculated in each case. (**B**) Average percentage of tumor debulked from mice 6 weeks post ip injection of control and chemotherapy treated HEY cells. The average tumor weight was standardised to total mouse body weight. Data has been extrapolated from a minimum of n = 6 mice in each group. Significant increase in tumor burden in cisplatin and paclitaxel treated HEY cell derived tumors compared to control untreated group, *P < 0.05. Images represent tumors debulked from one mouse in each group.

**Figure 7:**
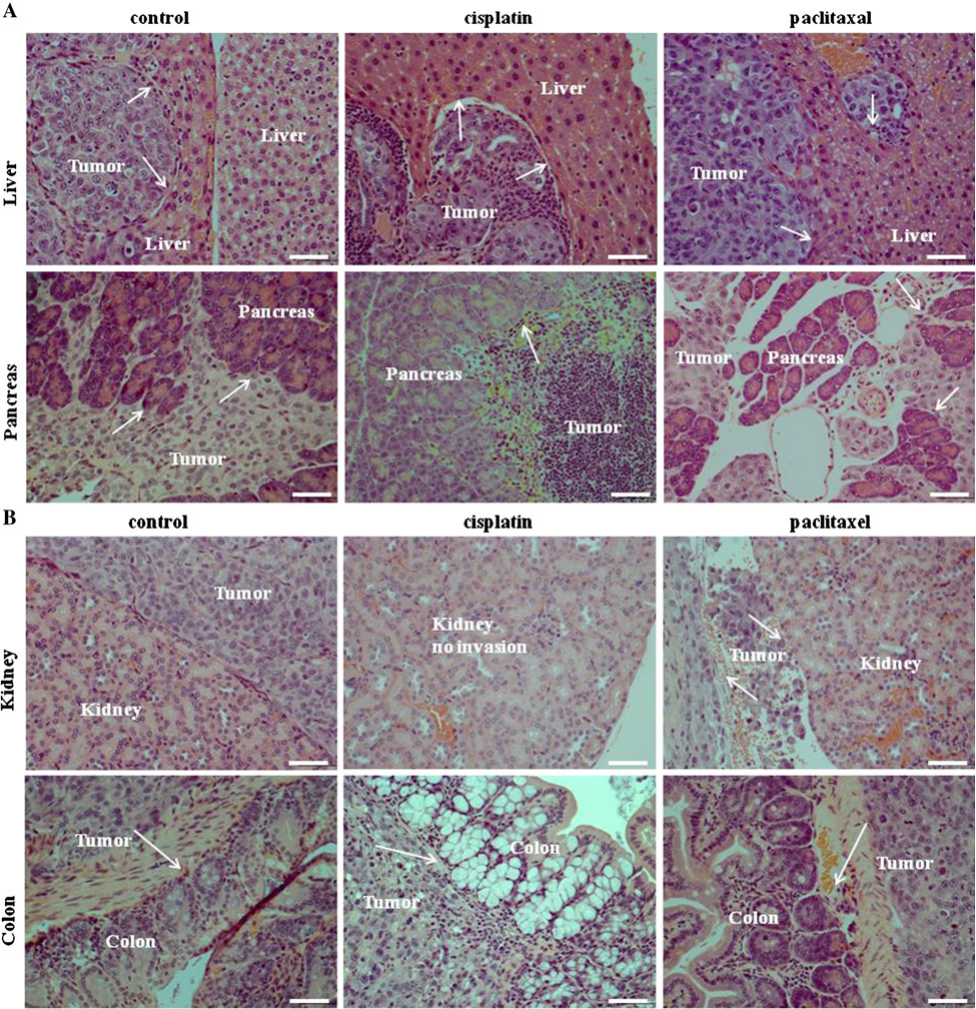
**H and E staining of control and chemotherapy treated HEY cell derived tumor associated infiltrated organs in mice.** 5 x 10^6^ cells were injected ip in each mouse. (**A**) Histological images of liver and pancreas showing infiltration of control and chemotherapy treated HEY cells in mice. (**B**) Histological images of mice kidney and colon injected with control and chemotherapy treated cells. Control cells surround the kidney with no invasion. Cisplatin treated cells do not invade kidney. Arrows indicate tumor cells invading the respective organs. Magnification 200X, scale bar = 10 μm.

In order to assess if the residual cancer cells after chemotherapy treatment retain tumorigenic abilities, an *in vivo* mouse intra-peritoneal (ip) HEY xenograft model was established. Five out of six mice injected with untreated HEY cells developed solid tumors in the form of 3–4 small lesions (<0.5 cm^3^) in the peritoneum within six to eight weeks. Tumors weighing 4.7% ± 1.1 of the total body weight were observed in all five cases (Figure 6). All twelve mice injected with the same number (5×10^6^) of cisplatin or paclitaxel treated cells (n = 6 in each group) developed tumors at the same time as control untreated cells, but with significantly enhanced tumor burden, being almost double that seen for cisplatin treated (8.7% ± 2.1 of the total body weight) and three-fold that of paclitaxel treated cells (13.32% ± 2.3 of the total body weight) (Figure 6). H & E staining of tumor infiltrated organs generated by control and treated cells showed the epithelial morphology of the cells infiltrating the abdominal organs (Figure 7). Injected control cells in mouse infiltrated liver, pancreas, stomach and colon but surrounded the kidney with no invasion (Figure 7A-B). Invasion into the liver and pancreas was common for cisplatin and paclitaxel treated injected cells (Figure 7A). Paclitaxel-treated HEY cells invaded kidney, but the invasion with the cisplatin treated cells was not consistent and differed between mice. In two out of the three mice analysed invasion to the kidney was observed, but in one mouse, cells surrounded the kidney with no invasion (Figure 7B).

**Figure 8:**
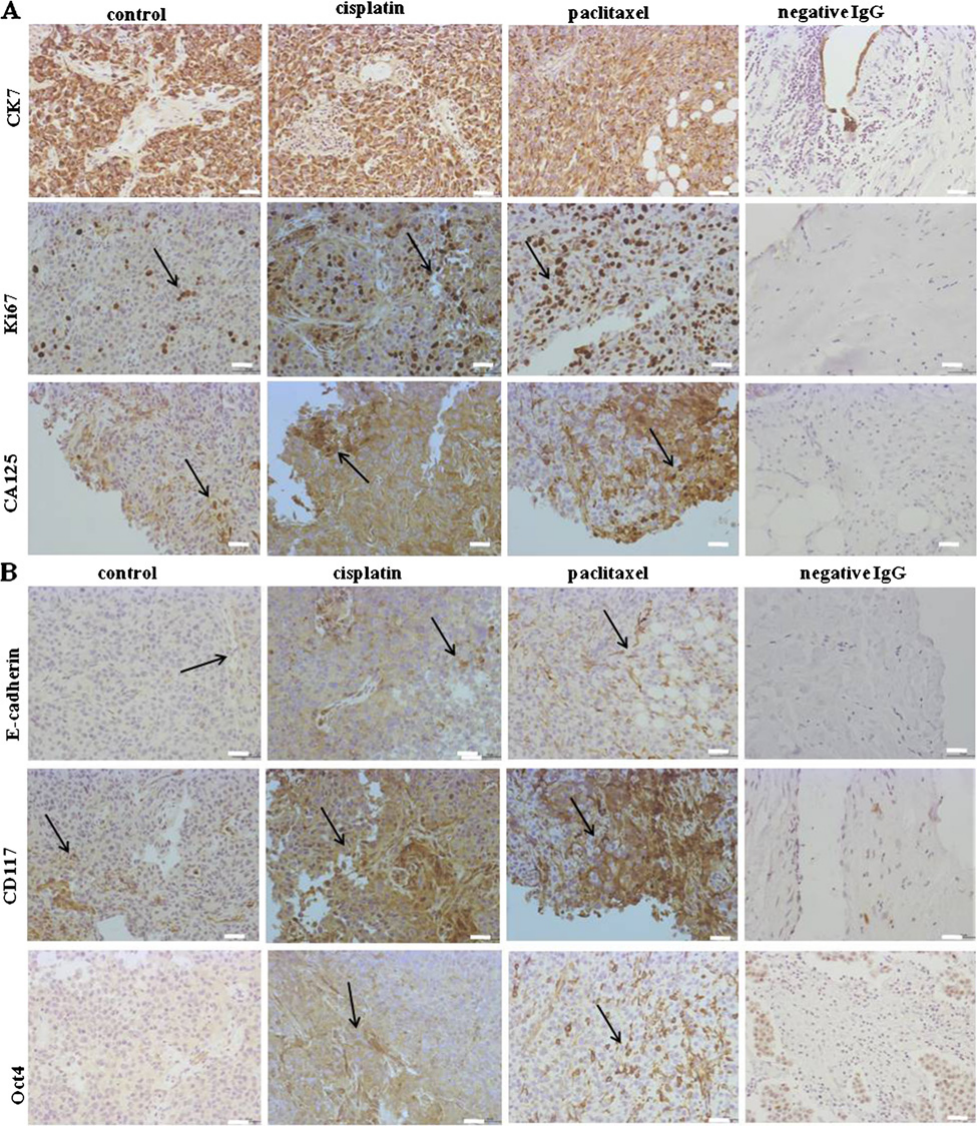
**(A-B) Immunohistochemistry images of mouse tumors generated from ip transplantation of control, cisplatin and paclitaxel-treated HEY cells.** Tumor sections were stained with antibodies specific for cyt7, Ki67, CA125, E-cadherin, CD117 and Oct4 as described in the Methods and material section. Magnification 200X, scale bar = 10 μm. Black arrows indicate specific antigen expression in respective tumor sections.

Immunohistochemistry analysis of mouse tumors demonstrated positive staining of cyt 7 in xenografts from both untreated and treated HEY cells (Figure 8A). Mouse xenografts also exhibited positive staining for Ki67, which was enhanced in cisplatin and paclitaxel treated cell-derived xenografts compared to untreated control xenografts (Figure 8A). Patches of E-cadherin staining localized to discrete cell-cell junctions were observed in untreated HEY xenografts (Figure 8B). This pattern of staining was enhanced in cisplatin and paclitaxel treated cell derived mouse xenografts (Figure 8B). A similar pattern of enhanced staining of CA125 was evident in treated cell mouse xenografts, compared to xenografts obtained from mice injected with untreated cells (Figure 8A). Mouse xenografts were also assessed for the expression of stem cells marker CD117 (c-Kit) and the embryonic stem cell marker Oct4. A dramatic increase in the expression of these two markers was observed in xenografts derived from cisplatin or paclitaxel treated cells, compared to the xenografts derived from control cells (Figure 8B).

## Discussion

Chemoresistance is a major obstacle towards the successful treatment of ovarian cancer patients. The molecular and the cellular mechanisms of the resistance of ovarian cancer cells to platinum and taxane-based therapies, the two agents used as standard treatment, remains unknown *in vitro* and *in vivo*. In this study we have used two very different ovarian cancer cell lines, OVCA 433 (mainly epithelial) and HEY (mainly mesenchymal), treated short-term with cisplatin or paclitaxel or the combination of both to dissect those initial cellular responses that facilitate the survival of residual cells and their subsequent regrowth in an *in vivo* mouse model. We have demonstrated that cisplatin or paclitaxel or combination treatment of ovarian cancer cell lines, generates in each case a population of residual cells with features of CSC-like cells. An enhanced expression of CSC markers in the residual cancer cells after chemotherapy treatments coincided with an enhanced expression of ERCC1 and/or β-tubulin isotype III, the two proteins commonly associated with resistance of cancer cells to platinum and taxane-based chemotherapies [29,30]. Enhancement in ERCC1 expression in response to cisplatin was consistent with the enhanced expression of β-tubulin isotype III within the same population of cells after paclitaxel treatment. However, in response to paclitaxel and combination treatments a greater degree of β-tubulin isotype III expression was observed, suggesting that cisplatin resistant cells may be cross resistant to paclitaxel but the reverse may not be the case. ERCC1 has been associated with cisplatin resistance in ovarian tumors and cancer cell lines [25,29]. Recent clinical trials suggest that patients with low ERCC1 levels benefit preferentially from cisplatin-based chemotherapy compared to patients who have a higher expression of ERCC1 in their tumors [31]. On the other hand, tumors resistant to paclitaxel or cancer cell lines rendered resistant to paclitaxel have substantially enhanced levels of isotypes III or IV β-tubulin [32-34]. Evidence for the enhancement in isotype-specific taxane-resistant tubulin has also been described in the tumors of ovarian cancer patients [26]. Paired samples from advanced-stage ovarian cancer patients who developed clinical paclitaxel resistance showed increases in β-tubulin isotypes I (3.6-fold), III (4.4-fold) and IV (7.6-fold) [26].

Long-term repeated chemo-treatment approaches have been shown to generate chemoresistant cancer cell lines with features of CSCs [35,36]. The novelty of the current study is the demonstration of CSC-like features in ovarian cancer cell lines by a single short-term exposure of chemotherapeutic agents. The fact that short-term single exposure of chemotherapeutic agents is capable of modulating the expression of specific chemoresistant genes (ERCC1 and β-tubulin III) and potential CSC genes, suggests that selection of existing chemoresistant CSC-like subpopulation of ovarian cancer cells is embedded within the bulk of the original cancer population. As shown in our previous studies, this pattern of selection of CSC-like cells is not limited to ovarian cancer cell lines but can be displayed in tumor cells isolated from primary ovarian tumors and ascites of ovarian cancer patients [11]. This suggests that in the clinical scenario, CSC enriched residual cells are generated in the host tumor microenvironment after the first round of chemotherapy treatment. Whether these cells further enrich their CSC-like characteristics after consecutive chemotherapy treatments or retain the original CSC-like features to facilitate the re-growth of secondary tumors is not known. However, we have previously demonstrated that the expression level of CSC-like markers in OVCA 433 cells remains unchanged after single or long-term treatments with cisplatin [12]. In this context, few previous studies have demonstrated the existence of CSC-enriched side population of cells [28,37] or CD44, CD117, CD133, CD24 enriched population of cells in ovarian cancer cell lines or ovarian cancer patient’s samples [38-40]. These CSC-enriched cells have been shown to develop tumors on sequential inoculation in nude mice and retain the original CSC-like phenotype observed in the parental sample.

Recent data suggest that CSCs rely on the presence of a ‘CSC niche’ which controls their self-renewal and differentiation [41]. Current studies have also shown that residual cells after chemotherapy treatment secrete soluble factors that provide a favourable microenvironment to facilitate the growth of residual cells [42,43]. This close relationship between chemotherapy-surviving cells and their secretory microenvironment represents a potential ‘CSC niche’ that can provide survival signals to residual cells for re-growth into a recurrent cancer. Moreover, CSCs can also be generated by the complex tumor microenvironment composed of diverse stromal cells, including tissue specific fibroblasts, cancer associated fibroblasts, tissue specific and bone marrow-derived mesenchymal stem cells, infiltrating immune cells, endothelial cells and their associated vascular network, soluble and other growth factors and/or extracellular matrix component [41]. Growth of recurrent tumors seems to rely on the permissive microenvironment provided by each component of ‘CSC niche’. The CSCs retain their exclusive abilities to self-renew and give rise to differentiated progenitor cells, while staying in an undifferentiated state themselves [41].

In the current study we have demonstrated that both the epithelial OVCA 433 and mesenchymal HEY cell lines respond to cisplatin or paclitaxel by enhancing the expression of CD24, CD117, CD133 and EpCAM. However, the enhancement of CD44 in response to cisplatin or paclitaxel treatments differed between the cell lines and may depend on the inherent epithelial or mesenchymal phenotype of the cell line. CD44 is not only a stem cell marker but has been shown to be highly expressed in cells with mesenchymal phenotype. The HEY cell line is inherently mesenchymal, with high endogenous expression of CD44 prior to chemotherapy. On the other hand, OVCA 433 is an epithelial cell line with a minimal expression of CD44. The addition of cisplatin drives both the cell lines to a mesenchymal state [12]. This correlates nicely with a slight increase in the expression of CD44 in both OVCA 433 and HEY cell lines. On the contrary, paclitaxel treatment induced a more epithelial-like morphology in the inherently mesenchymal HEY cell line, which may result in the down regulation of CD44 expression. This holds true only at the protein level. At the mRNA level, the expression of CD44 was elevated with all chemotherapy treatments in both the cell lines. This suggests, an inability of translation of CD44 mRNA in HEY cells. This may occur due to epigenetic changes in CD44 with paclitaxel treatment in HEY cells [44]. However, the disparity of EpCAM expression at the protein and mRNA levels in HEY cells is difficult to explain. One possible explanation can be that DNA damage response initiated by cisplatin has no effect on the transcriptional expression of EpCAM but it may trigger enhanced translation of EpCAM from the existing endogenous EpCAM mRNA.

Tumors generated from control untreated and cisplatin/paclitaxel treated cells were invasive and invaded peritoneal organs such as pancreas and liver. With the small number of tumor xenografts analysed in this study (n = 3) we have demonstrated some differences in the invasion to kidney by chemotherapy treated cells. No pattern of kidney invasion was observed with control untreated mice. However, paclitaxel-treated HEY cells invaded kidney, but the invasion with cisplatin treated cells was not consistent and differed between mice. In two out of the three mice analysed, invasion to kidney was observed, but in one mouse tumor cells surrounded the kidney with no apparent invasion. This variation in the invasion pattern between the control and chemotherapy treated cells may be due to the phenotypic changes induced in the cells by the chemotherapeutic agents or it may be due to the induced ‘CSC-niche’ created by the cells within the tumor microenvironment.

Enhanced CSC-like characteristics observed in ovarian cancer cells after a single dose of chemotherapy treatment were retained in *in vivo* mouse xenografts (enhanced expression of Oct4 and CD117 in tumors derived from cisplatin and paclitaxel treated cells). Tumor cells within the xenografts of chemotherapy treated cells had a greater proliferative potential as evaluated by enhanced Ki67 staining, and a greater tumor burden within the same time frame as that of the tumors derived from control untreated cells. In addition, tumors derived from chemotherapy treated cells had an enhanced expression of CA125 and were more epithelial in phenotype with enhanced E-cadherin expression compared to tumors generated from control untreated HEY cells. The relative high abundance of epithelial markers (enhanced expression of E-cadherin and CA125) in tumors derived from HEY cells treated with chemotherapy *in vitro,* compared to untreated control cells, is consistent with our recent observation of ascites tumor cells of recurrent patients which had an enhanced expression of epithelial and CSC-like markers compared to tumor cells of ascites obtained from chemonaive untreated patients [13]. We have previously reported that ovarian cancer cells possess a certain level of epithelial mesenchymal plasticity that allows them to change their phenotype and acquire different functions and properties under the influence of the local tumor environment [12,45,46]. Considering that HEY cells have inherent mesenchymal phenotype and very low/no expression of E-cadherin and CA125 *in vitro*, the expression of E-cadherin and CA125 *in vivo* control mouse xenografts implies such plasticity. The dynamics of ovarian tumor cell plasticity in relation to tumor cell dissemination and engraftment on secondary site is not well understood but the potential ‘mesenchymal to epithelial transition’ (MET) is assumed to occur in the late phase of ovarian tumor dissemination when the tumor cells adapt to the ascites microenvironment [46-49]. The expression of E-cadherin and CA125 in xenografts obtained from mesenchymal HEY cells, and enhancement of that expression in mouse xenografts derived from residual chemotherapy treated cells, further illustrates plasticity related changes in HEY cells influenced by the *in vivo* microenvironment which acts as a ‘CSC niche’, and may facilitate the rapid proliferation of chemotherapy-treated CSC-rich residual cells resulting in increased tumor burden. These novel observations are consistent with a recent study that demonstrated the epithelial phenotype of side population cells sorted from ovarian cancer lines and ascites of ovarian cancer patients [50]. These stem-like side population cells exhibited decreased adhesive and invasive potential compared to the more differentiated non-side population cells and were localized on tumor boundary when implanted into nude mice along with non-side population cells [50]. These results suggest that the relationship between malignant potential, CSC phenotype and cellular plasticity in ovarian cancer is a developing field and more research is needed to understand the processes. In this context, the identification of E-cadherin rich metastatic tumors in breast and brain cancers [48,51,52], and an association between increased pluripotency and the epithelial subcomponent of human bladder and prostatic carcinoma cells [53], and normal breast cells [54] exerts a strong link between epithelial plasticity and CSCs. Perhaps consistent with this is the observation that BRCA1-associated basal breast cancers better resemble aberrant luminal progenitor cells rather than the mesenchymal-like mammary stem cells [55,56].

**Figure 9:**
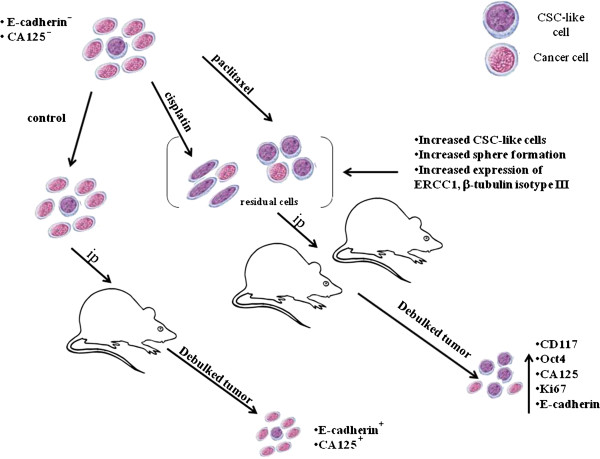
**Mouse model of chemoresistance and associated recurrence in ovarian cancer.** Control untreated and residual HEY cells after treatment with cisplatin or paclitaxel *in vitro* were injected (ip) into nude mice (n = 18, n = 6/group) and followed for 5–7 weeks. Cisplatin and paclitaxel treated cells enriched in CSC-like markers generated significantly increased tumor burden as well as xenografts with enhanced expression of CD117, Oct4, CA125, Ki67 and E-cadherin compared to tumors derived from non treated HEY cells. This suggests that chemotherapy treatment promotes CSC-dependent enhanced tumor progression in a mouse model of ovarian cancer.

The results from this novel study show that (a) a short-term early phase chemotherapy treatment leaves residual cells that are enriched for CSC-like traits, (b) in an *in vivo* environment, these cells are more proliferative and result in a larger tumor burden, and (c) the cells retain the CSC enriched phenotype in the resultant tumors. These findings are strikingly similar to ovarian cancer patients who relapse post-chemotherapy treatment with increased tumor burden and metastasis with recurrent tumors that are enriched for CSC-like traits [13,14]. On the basis of our novel findings a model of chemoresistance and recurrence in ovarian carcinomas is described in Figure 9.

## Competing interests

The authors declare that they have no competing interests.

## Authors’ contributions

KA and NA conceived the idea and designed the experiments; KA, AL and RL performed the experiments; MAQ, EWT and JFK edited the manuscript; SN analyzed the data for organ invasion, HZ provided tools to perform experiments. All authors read and approved the manuscript.

## Supplementary Material

Additional file 1: Figure S1 Expression of chemoresistant phenotype in OVCA 433 cell line. Expression and immunolocalization of (A) ERCC1 and (B) β-tubulin isotype III in OVCA 433 cell line in response to cisplatin, paclitaxel and combination treatment. The images were evaluated as described in Figure 2. Click here for file

Additional file 2: Figure S2 The effects of cisplatin, paclitaxel and combination treatments on the expression of CSC-like markers in OVCA 433 cells. The experiment was performed as described in Figure 3. Click here for file

Additional file 3: Figure S3 mRNA expression of EpCAM, Nanog, CD44, CD117 and Oct4 in OVCA 433 cell line in response to chemotherapy treatments (cisplatin, paclitaxel and combination). The experiment was performed as described in Figure 4. Click here for file

Additional file 4: Figure S4 Effects of chemotherapy on the sphere forming ability of OVCA 433 cells. The sphere-forming assay was performed on low attachment plates as described in figure 5. Significantly different in the chemotherapy treated cells compared to control untreated cells. *P<0.05, ** P<0.01. Click here for file
