# Supplementary figures and images for: Short-term single treatment of chemotherapy results in the enrichment of ovarian cancer stem cell-like cells leading to an increased tumor burden
Source: Mol Cancer. 2013 Mar 27;12:24. doi: 10.1186/1476-4598-12-24 (PMC3668985; doi:10.1186/1476-4598-12-24)

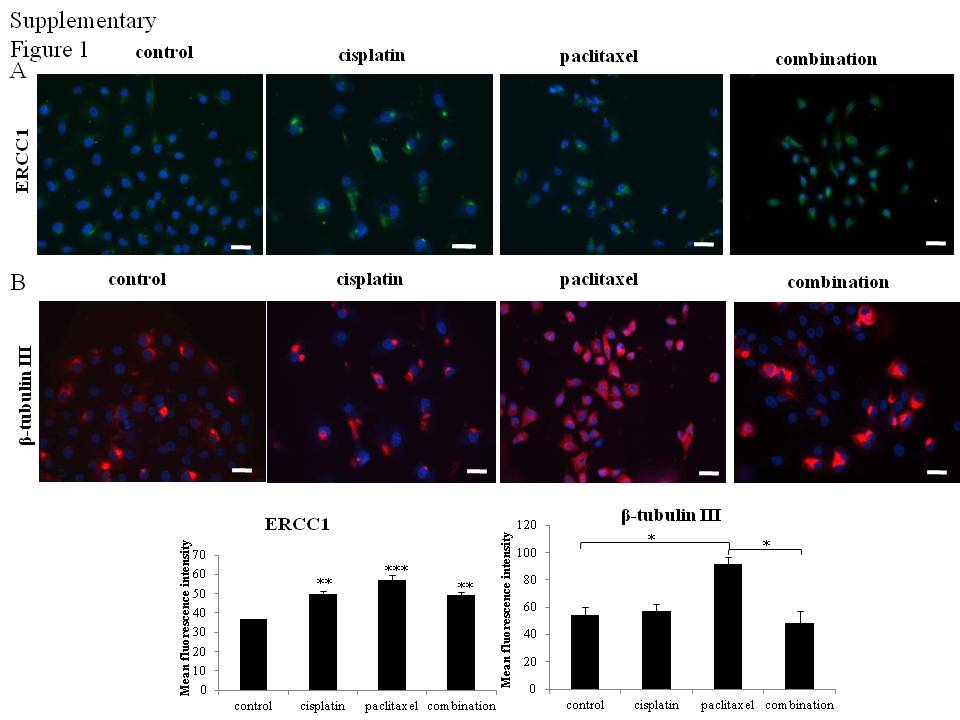

Supplement: Additional file 1: Figure S1 — Expression of chemoresistant phenotype in OVCA 433 cell line. Expression and immunolocalization of (A) ERCC1 and (B) β-tubulin isotype III in OVCA 433 cell line in response to cisplatin, paclitaxel and combination treatment. The images were evaluated as described in Figure 2. [file 1476-4598-12-24-S1.jpeg]

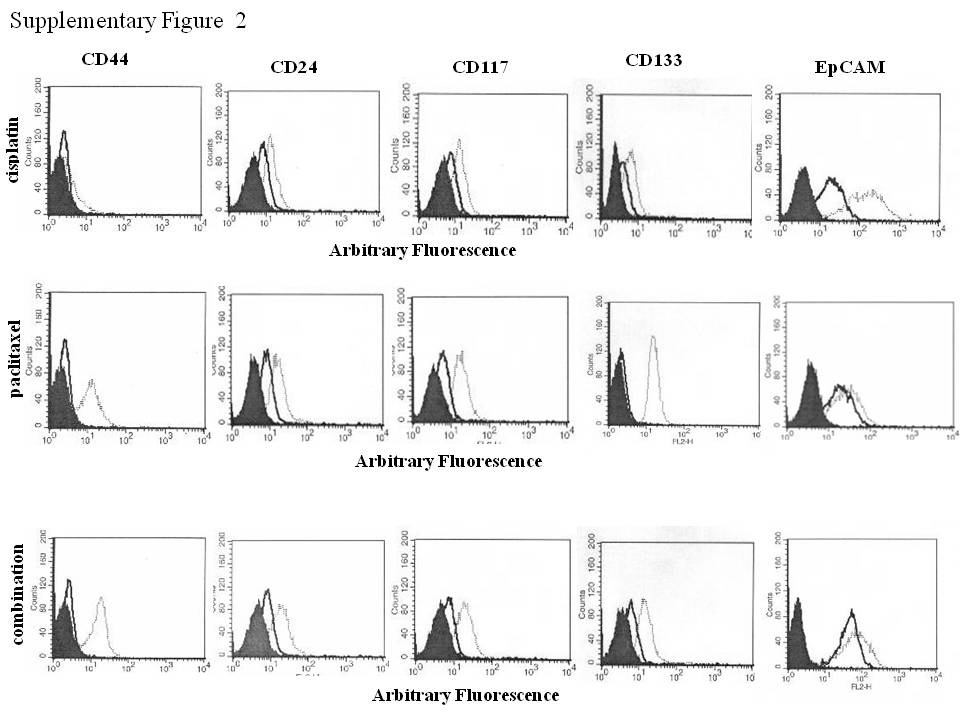

Supplement: Additional file 2: Figure S2 — The effects of cisplatin, paclitaxel and combination treatments on the expression of CSC-like markers in OVCA 433 cells. The experiment was performed as described in Figure 3. [file 1476-4598-12-24-S2.jpeg]

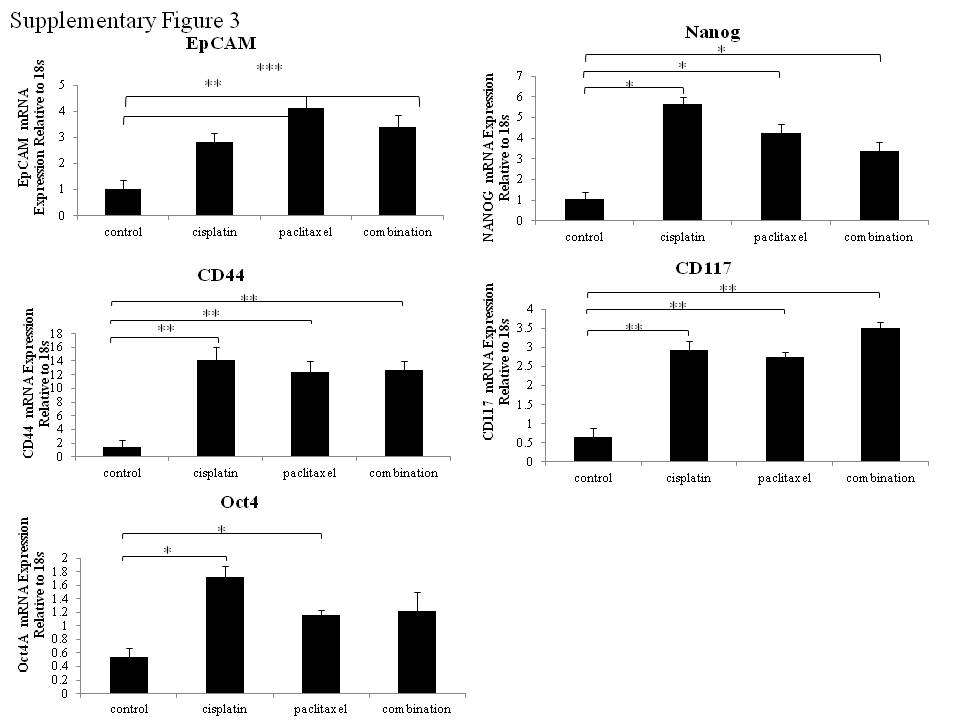

Supplement: Additional file 3: Figure S3 — mRNA expression of EpCAM, Nanog, CD44, CD117 and Oct4 in OVCA 433 cell line in response to chemotherapy treatments (cisplatin, paclitaxel and combination). The experiment was performed as described in Figure 4. [file 1476-4598-12-24-S3.jpeg]

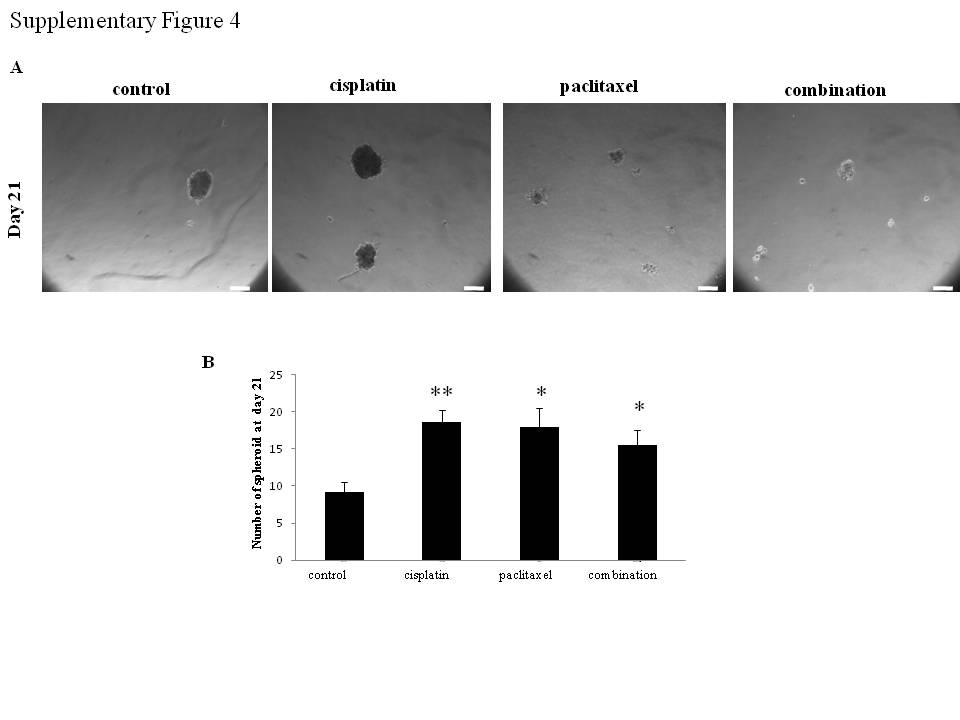

Supplement: Additional file 4: Figure S4 — Effects of chemotherapy on the sphere forming ability of OVCA 433 cells. The sphere-forming assay was performed on low attachment plates as described in figure 5. Significantly different in the chemotherapy treated cells compared to control untreated cells. *P<0.05, ** P<0.01. [file 1476-4598-12-24-S4.jpeg]
